# Crystal structure of (2*E*)-*N*-methyl-2-[(4-oxo-4*H*-chromen-3-yl)methyl­idene]hydrazine­carbo­thio­amide

**DOI:** 10.1107/S1600536814021667

**Published:** 2014-10-08

**Authors:** G. Vimala, J. Govindaraj, J. Haribabu, R. Karvembu, A. SubbiahPandi

**Affiliations:** a Department of Physics, Presidency College (Autonomous), Chennai 600 005, India; b Department of Physics, Pachaiyappa’s College for Men, Kancheepuram 631 501, India; c Department of Chemistry, National Institute of Technology, Trichy 620 015, India; d Deparment of Chemistry, National Institute of Technology, Trichy 620 015, India

**Keywords:** crystal structure, hydrazinecarbo­thio­amide, 4*H*-chromen-4-one, biological properties, hydrogen bonding

## Abstract

In the title compound, C_12_H_11_N_3_O_2_S, the dihedral angle between the 4*H*-chromen-4-one ring system and the –CH=N—NH—CS—NH– unit is 6.22 (1)°. In the crystal, inversion dimers linked by pairs of N—H⋯O hydrogen bonds generate *R*_2_^2^(14) loops. The dimers are reinforced by a pair of C—H⋯O inter­actions, which generate *R*_2_^2^(10) loops.

## Related literature   

For the biological properties of related compounds, see: Khan *et al.* (2009 ▶); Tu *et al.* (2013 ▶); Kelly *et al.* (1996 ▶). For a related structure, see: Ishikawa & Watanabe (2014 ▶).
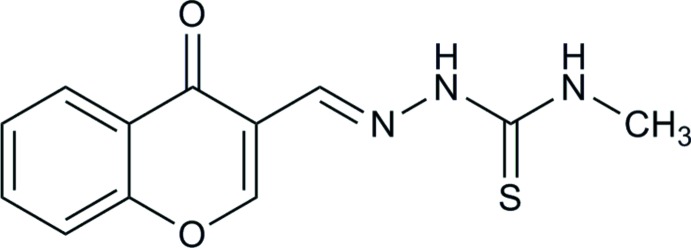


## Experimental   

### Crystal data  

C_12_H_11_N_3_O_2_S
*M* *_r_* = 261.30
Monoclinic, 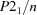

*a* = 6.3702 (7) Å
*b* = 20.647 (2) Å
*c* = 9.2717 (10) Å
β = 98.365 (3)°
*V* = 1206.5 (2) Å^3^
*Z* = 4
Mo *K*α radiation
μ = 0.27 mm^−1^
*T* = 293 K
0.30 × 0.25 × 0.20 mm

### Data collection  

Bruker SMART APEXII CCD diffractometer
Absorption correction: multi-scan (*SADABS*; Bruker, 2008 ▶) *T*_min_ = 0.924, *T*_max_ = 0.948
17429 measured reflections
3560 independent reflections
2257 reflections with *I* > 2σ(*I*)
*R*_int_ = 0.031

### Refinement  

*R*[*F* ^2^ > 2σ(*F* ^2^)] = 0.043
*wR*(*F* ^2^) = 0.131
*S* = 1.06
3560 reflections
164 parameters
H-atom parameters constrained
Δρ_max_ = 0.22 e Å^−3^
Δρ_min_ = −0.24 e Å^−3^

### 

Data collection: *APEX2* (Bruker, 2008 ▶); cell refinement: *SAINT* (Bruker, 2008 ▶); data reduction: *SAINT*; program(s) used to solve structure: *SHELXS97* (Sheldrick, 2008 ▶); program(s) used to refine structure: *SHELXL97* (Sheldrick, 2008 ▶); molecular graphics: *ORTEP-3 for Windows* (Farrugia, 2012 ▶); software used to prepare material for publication: *SHELXL97* and *PLATON* (Spek, 2009 ▶).

## Supplementary Material

Crystal structure: contains datablock(s) global, I. DOI: 10.1107/S1600536814021667/hb7287sup1.cif

Structure factors: contains datablock(s) I. DOI: 10.1107/S1600536814021667/hb7287Isup2.hkl

Supporting information file. DOI: 10.1107/S1600536814021667/hb7287Isup3.cml

**Fig. 1.**
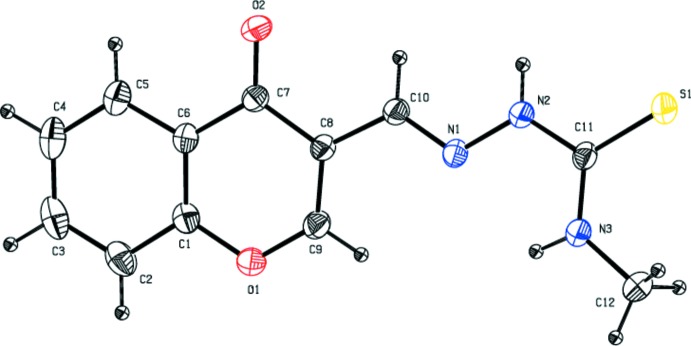
The molecular structure of the title compound, with displacement ellipsoids drawn at the 30% probability level.

. DOI: 10.1107/S1600536814021667/hb7287fig1.tif
The mol­ecular structure of the title compound, with displacement ellipsoids drawn at the 30% probability level.

**Fig. 2.**
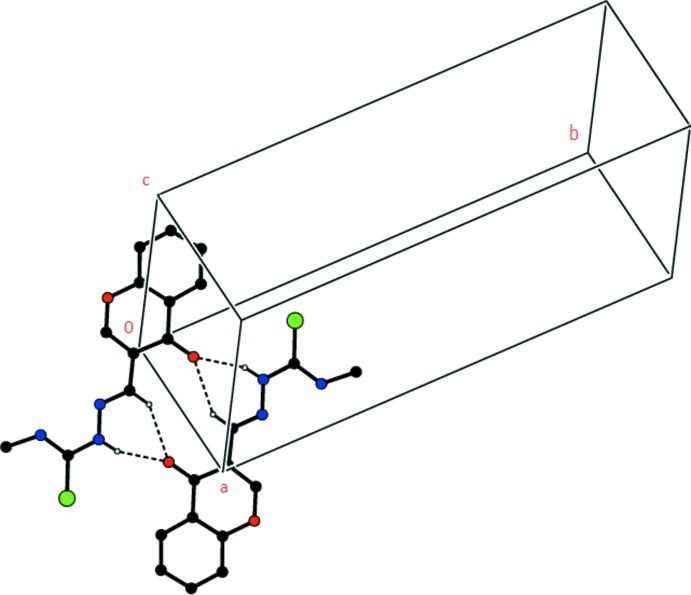
The packing of the title compound with hydrogen bonds represented by dashed lines. Hydrogen atoms not involved in these bonds are omitted for clarity.

. DOI: 10.1107/S1600536814021667/hb7287fig2.tif
The packing of the title compound with hydrogen bonds represented by dashed lines. Hydrogen atoms not involved in these bonds are omitted for clarity.

CCDC reference: 1027156

Additional supporting information:  crystallographic information; 3D view; checkCIF report

## Figures and Tables

**Table 1:** Hydrogen-bond geometry (, )

*D*H*A*	*D*H	H*A*	*D* *A*	*D*H*A*
N2H2*A*O2^i^	0.86	2.11	2.897(2)	152
C10H10O2^i^	0.93	2.44	3.219(2)	141

Symmetry code: (i) 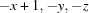.

## supplementary crystallographic information

### S1. Comment

Thiosemicarbazones are of considerable interest because of their versatile chemistry and various biological activites such as antitumor, antibacterial, antiviral, antiamoebic and antimalarial (Kelly *et al.*, 1996). Schiff bases derived from 3-formylchromones have attracted much attention due to their biological functions such as enzyme inhibition (Khan *et al.*, 2009; Tu *et al.*, 2013).

The structure of the title compound (Figure 1) shows that the atoms of both 4*H*-chromen-4-one and the –CH=N—NH—CS—NH– segments are roughly planar and the largest deviations are -0.144 (2) and -0.114 (2) Å for O2 and C12 respectively. The dihedral angles between 4*H*-chromen-4-one and –CH=N—NH—CS—NH—C– unit and the benzene ring of 4*H*-chromen-4-one and –CH=N—NH—CS—NH—C– unit are 6.22 (1) and 7.12 (1)°, respectively.

In the crystal, inversion dimers linked by pairs of N—H···O hydrogen bonds generate R_2_^2^(14) loops. The dimers are reinforced by a pair of C—H···O interactions, which generate R_2_^2^(10) loops.

### S2. Experimental

1.05 g (0.01 mol) of *N*-methylhydrazinecarbothioamide was dissolved in 20 ml of hot ethanol and to this 1.74 g of 4-oxo-4*H*-Chromene-3-carbaldehydein 10 ml of ethanol was added and continuously stirred for a period of 10 min with continuous stirring. The reaction mixture was refluxed for 2 h and allowed to cool whereby shining white was filtered and washed thoroughly with ethanol and then dried in vaccum. The compound was recrystallized from hot ethanol to yield colourless blocks in 92% yield.

### S3. Refinement

All H atoms were fixed geometrically and allowed to ride on their parent C atoms, with C—H distances fixed in the range 0.93–0.97 Å with *U*_iso_(H) = 1.5*U*_eq_(C) for methyl H atoms and 1.2*U*_eq_(C) for all other H atoms.

### Crystal data

**Table:**

C_12_H_11_N_3_O_2_S	*Z* = 4
*M**_r_* = 261.30	*F*(000) = 544
Monoclinic, *P*2_1_/*n*	*D*_x_ = 1.439 Mg m^−^^3^
Hall symbol: -P 2yn	Mo *K*α radiation, λ = 0.71073 Å
*a* = 6.3702 (7) Å	µ = 0.27 mm^−^^1^
*b* = 20.647 (2) Å	*T* = 293 K
*c* = 9.2717 (10) Å	Block, colourless
β = 98.365 (3)°	0.30 × 0.25 × 0.20 mm
*V* = 1206.5 (2) Å^3^	

### Data collection

**Table:**

Bruker SMART APEXII CCD diffractometer	3560 independent reflections
Radiation source: fine-focus sealed tube	2257 reflections with *I* > 2σ(*I*)
Graphite monochromator	*R*_int_ = 0.031
ω and φ scans	θ_max_ = 30.2°, θ_min_ = 2.0°
Absorption correction: multi-scan (*SADABS*; Bruker, 2008)	*h* = −8→8
*T*_min_ = 0.924, *T*_max_ = 0.948	*k* = −28→28
17429 measured reflections	*l* = −12→12

### Refinement

**Table:**

Refinement on *F*^2^	Primary atom site location: structure-invariant direct methods
Least-squares matrix: full	Secondary atom site location: difference Fourier map
*R*[*F*^2^ > 2σ(*F*^2^)] = 0.043	Hydrogen site location: inferred from neighbouring sites
*wR*(*F*^2^) = 0.131	H-atom parameters constrained
*S* = 1.06	*w* = 1/[σ^2^(*F*_o_^2^) + (0.056*P*)^2^ + 0.3687*P*] where *P* = (*F*_o_^2^ + 2*F*_c_^2^)/3
3560 reflections	(Δ/σ)_max_ < 0.001
164 parameters	Δρ_max_ = 0.22 e Å^−^^3^
0 restraints	Δρ_min_ = −0.24 e Å^−^^3^

### Special details

**Table:**

Geometry. All e.s.d.'s (except the e.s.d. in the dihedral angle between two l.s. planes) are estimated using the full covariance matrix. The cell e.s.d.'s are taken into account individually in the estimation of e.s.d.'s in distances, angles and torsion angles; correlations between e.s.d.'s in cell parameters are only used when they are defined by crystal symmetry. An approximate (isotropic) treatment of cell e.s.d.'s is used for estimating e.s.d.'s involving l.s. planes.
Refinement. Refinement of *F*^2^ against ALL reflections. The weighted *R*-factor *wR* and goodness of fit *S* are based on *F*^2^, conventional *R*-factors *R* are based on *F*, with *F* set to zero for negative *F*^2^. The threshold expression of *F*^2^ > σ(*F*^2^) is used only for calculating *R*-factors(gt) *etc*. and is not relevant to the choice of reflections for refinement. *R*-factors based on *F*^2^ are statistically about twice as large as those based on *F*, and *R*- factors based on ALL data will be even larger.

### Fractional atomic coordinates and isotropic or equivalent isotropic displacement parameters (Å^2^)

**Table:**

	*x*	*y*	*z*	*U*_iso_*/*U*_eq_	
C1	1.1216 (3)	−0.01086 (8)	−0.30947 (18)	0.0402 (4)	
C2	1.2628 (3)	−0.03414 (11)	−0.3973 (2)	0.0536 (5)	
H2	1.3795	−0.0097	−0.4138	0.064*	
C3	1.2268 (4)	−0.09395 (12)	−0.4591 (2)	0.0615 (6)	
H3	1.3198	−0.1102	−0.5187	0.074*	
C4	1.0550 (4)	−0.13046 (11)	−0.4344 (2)	0.0590 (6)	
H4	1.0321	−0.1708	−0.4784	0.071*	
C5	0.9167 (3)	−0.10774 (9)	−0.3453 (2)	0.0485 (5)	
H5	0.8020	−0.1329	−0.3279	0.058*	
C6	0.9493 (3)	−0.04658 (8)	−0.28081 (17)	0.0359 (4)	
C7	0.8095 (3)	−0.02066 (8)	−0.18335 (17)	0.0353 (4)	
C8	0.8552 (3)	0.04523 (7)	−0.13502 (17)	0.0336 (3)	
C9	1.0264 (3)	0.07560 (8)	−0.1711 (2)	0.0429 (4)	
H9	1.0518	0.1177	−0.1376	0.051*	
C10	0.7153 (3)	0.07618 (8)	−0.04415 (18)	0.0373 (4)	
H10	0.5918	0.0552	−0.0274	0.045*	
C11	0.6662 (3)	0.21399 (7)	0.16614 (18)	0.0367 (4)	
C12	0.9249 (4)	0.30201 (10)	0.2112 (3)	0.0619 (6)	
H12A	0.8427	0.3380	0.1689	0.093*	
H12B	1.0703	0.3075	0.1976	0.093*	
H12C	0.9158	0.2998	0.3136	0.093*	
N1	0.7607 (2)	0.13156 (6)	0.01244 (15)	0.0367 (3)	
N2	0.6206 (2)	0.15691 (6)	0.09629 (16)	0.0400 (3)	
H2A	0.5048	0.1368	0.1046	0.048*	
N3	0.8432 (3)	0.24283 (7)	0.14142 (18)	0.0456 (4)	
H3A	0.9141	0.2252	0.0797	0.055*	
O1	1.1633 (2)	0.04988 (6)	−0.25169 (15)	0.0482 (3)	
O2	0.6644 (2)	−0.05230 (6)	−0.14399 (16)	0.0550 (4)	
S1	0.49924 (9)	0.24309 (2)	0.27366 (6)	0.05253 (18)	

### Atomic displacement parameters (Å^2^)

**Table:**

	*U*^11^	*U*^22^	*U*^33^	*U*^12^	*U*^13^	*U*^23^
C1	0.0460 (10)	0.0399 (9)	0.0355 (9)	0.0073 (7)	0.0089 (7)	0.0057 (7)
C2	0.0545 (12)	0.0624 (12)	0.0470 (11)	0.0127 (10)	0.0180 (9)	0.0078 (9)
C3	0.0724 (15)	0.0678 (14)	0.0469 (12)	0.0299 (12)	0.0167 (10)	0.0005 (10)
C4	0.0805 (16)	0.0484 (11)	0.0468 (11)	0.0189 (11)	0.0050 (11)	−0.0096 (9)
C5	0.0583 (12)	0.0403 (9)	0.0451 (10)	0.0052 (8)	0.0011 (9)	−0.0057 (8)
C6	0.0422 (9)	0.0336 (8)	0.0307 (8)	0.0050 (7)	0.0018 (7)	0.0015 (6)
C7	0.0365 (9)	0.0348 (8)	0.0339 (8)	−0.0016 (6)	0.0024 (7)	−0.0008 (6)
C8	0.0377 (9)	0.0302 (7)	0.0328 (8)	−0.0005 (6)	0.0052 (7)	0.0005 (6)
C9	0.0517 (11)	0.0329 (8)	0.0463 (10)	−0.0033 (7)	0.0147 (8)	0.0002 (7)
C10	0.0397 (9)	0.0353 (8)	0.0375 (9)	−0.0028 (7)	0.0080 (7)	−0.0028 (7)
C11	0.0430 (10)	0.0274 (7)	0.0401 (9)	0.0026 (6)	0.0072 (7)	0.0012 (6)
C12	0.0564 (13)	0.0406 (10)	0.0913 (17)	−0.0104 (9)	0.0190 (12)	−0.0151 (10)
N1	0.0445 (8)	0.0310 (7)	0.0357 (7)	0.0011 (6)	0.0097 (6)	−0.0006 (5)
N2	0.0427 (8)	0.0328 (7)	0.0469 (8)	−0.0036 (6)	0.0150 (7)	−0.0058 (6)
N3	0.0500 (9)	0.0330 (7)	0.0578 (10)	−0.0036 (6)	0.0213 (8)	−0.0072 (6)
O1	0.0510 (8)	0.0410 (7)	0.0572 (8)	−0.0043 (5)	0.0233 (6)	0.0007 (6)
O2	0.0539 (8)	0.0445 (7)	0.0712 (10)	−0.0181 (6)	0.0241 (7)	−0.0158 (6)
S1	0.0572 (3)	0.0399 (3)	0.0659 (4)	0.0015 (2)	0.0269 (3)	−0.0093 (2)

### Geometric parameters (Å, º)

**Table:**

C1—O1	1.374 (2)	C8—C10	1.460 (2)
C1—C6	1.380 (2)	C9—O1	1.338 (2)
C1—C2	1.384 (3)	C9—H9	0.9300
C2—C3	1.367 (3)	C10—N1	1.274 (2)
C2—H2	0.9300	C10—H10	0.9300
C3—C4	1.375 (3)	C11—N3	1.324 (2)
C3—H3	0.9300	C11—N2	1.356 (2)
C4—C5	1.375 (3)	C11—S1	1.6715 (17)
C4—H4	0.9300	C12—N3	1.444 (2)
C5—C6	1.400 (2)	C12—H12A	0.9600
C5—H5	0.9300	C12—H12B	0.9600
C6—C7	1.459 (2)	C12—H12C	0.9600
C7—O2	1.230 (2)	N1—N2	1.3695 (18)
C7—C8	1.449 (2)	N2—H2A	0.8600
C8—C9	1.342 (2)	N3—H3A	0.8600
			
O1—C1—C6	121.78 (15)	O1—C9—C8	124.97 (16)
O1—C1—C2	116.17 (17)	O1—C9—H9	117.5
C6—C1—C2	122.05 (17)	C8—C9—H9	117.5
C3—C2—C1	118.5 (2)	N1—C10—C8	120.64 (15)
C3—C2—H2	120.8	N1—C10—H10	119.7
C1—C2—H2	120.8	C8—C10—H10	119.7
C2—C3—C4	121.0 (2)	N3—C11—N2	115.89 (15)
C2—C3—H3	119.5	N3—C11—S1	124.97 (13)
C4—C3—H3	119.5	N2—C11—S1	119.14 (13)
C5—C4—C3	120.5 (2)	N3—C12—H12A	109.5
C5—C4—H4	119.7	N3—C12—H12B	109.5
C3—C4—H4	119.7	H12A—C12—H12B	109.5
C4—C5—C6	119.8 (2)	N3—C12—H12C	109.5
C4—C5—H5	120.1	H12A—C12—H12C	109.5
C6—C5—H5	120.1	H12B—C12—H12C	109.5
C1—C6—C5	118.20 (16)	C10—N1—N2	116.71 (14)
C1—C6—C7	119.82 (15)	C11—N2—N1	119.45 (14)
C5—C6—C7	121.97 (16)	C11—N2—H2A	120.3
O2—C7—C8	122.21 (15)	N1—N2—H2A	120.3
O2—C7—C6	122.79 (15)	C11—N3—C12	124.28 (16)
C8—C7—C6	115.00 (14)	C11—N3—H3A	117.9
C9—C8—C7	119.72 (15)	C12—N3—H3A	117.9
C9—C8—C10	121.73 (15)	C9—O1—C1	118.35 (14)
C7—C8—C10	118.52 (14)		
			
O1—C1—C2—C3	−179.09 (17)	C6—C7—C8—C9	−5.2 (2)
C6—C1—C2—C3	1.2 (3)	O2—C7—C8—C10	−4.0 (2)
C1—C2—C3—C4	−0.3 (3)	C6—C7—C8—C10	176.46 (14)
C2—C3—C4—C5	−0.8 (3)	C7—C8—C9—O1	0.6 (3)
C3—C4—C5—C6	0.9 (3)	C10—C8—C9—O1	178.80 (16)
O1—C1—C6—C5	179.29 (15)	C9—C8—C10—N1	−5.1 (3)
C2—C1—C6—C5	−1.0 (3)	C7—C8—C10—N1	173.16 (15)
O1—C1—C6—C7	−1.8 (2)	C8—C10—N1—N2	−179.90 (14)
C2—C1—C6—C7	177.85 (16)	N3—C11—N2—N1	3.0 (2)
C4—C5—C6—C1	−0.1 (3)	S1—C11—N2—N1	−177.53 (12)
C4—C5—C6—C7	−178.91 (17)	C10—N1—N2—C11	176.29 (15)
C1—C6—C7—O2	−173.71 (17)	N2—C11—N3—C12	−176.58 (18)
C5—C6—C7—O2	5.1 (3)	S1—C11—N3—C12	4.0 (3)
C1—C6—C7—C8	5.8 (2)	C8—C9—O1—C1	3.8 (3)
C5—C6—C7—C8	−175.36 (16)	C6—C1—O1—C9	−3.1 (2)
O2—C7—C8—C9	174.28 (17)	C2—C1—O1—C9	177.22 (16)

### Hydrogen-bond geometry (Å, º)

**Table:**

*D*—H···*A*	*D*—H	H···*A*	*D*···*A*	*D*—H···*A*
N2—H2*A*···O2^i^	0.86	2.11	2.897 (2)	152
C10—H10···O2^i^	0.93	2.44	3.219 (2)	141

Symmetry code: (i) −*x*+1, −*y*, −*z*.
