# Supplementary figures and images for: Crystal structure of (2E)-N-methyl-2-[(4-oxo-4H-chromen-3-yl)methyl­idene]hydrazine­carbo­thio­amide
Source: Acta Crystallogr Sect E Struct Rep Online. 2014 Oct 8;70(Pt 11):o1151. doi: 10.1107/S1600536814021667 (PMC4257262; doi:10.1107/S1600536814021667)

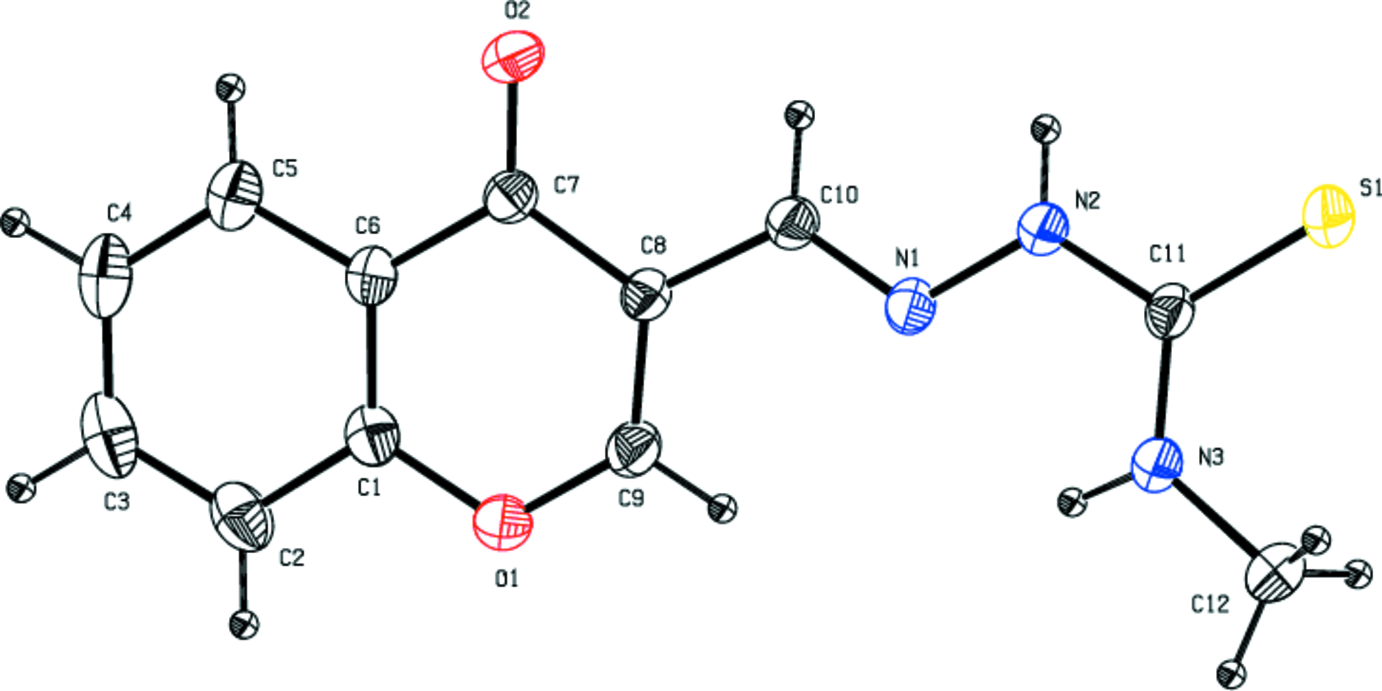

Supplement: Supplementary file 4 [file e-70-o1151-fig1.tif]

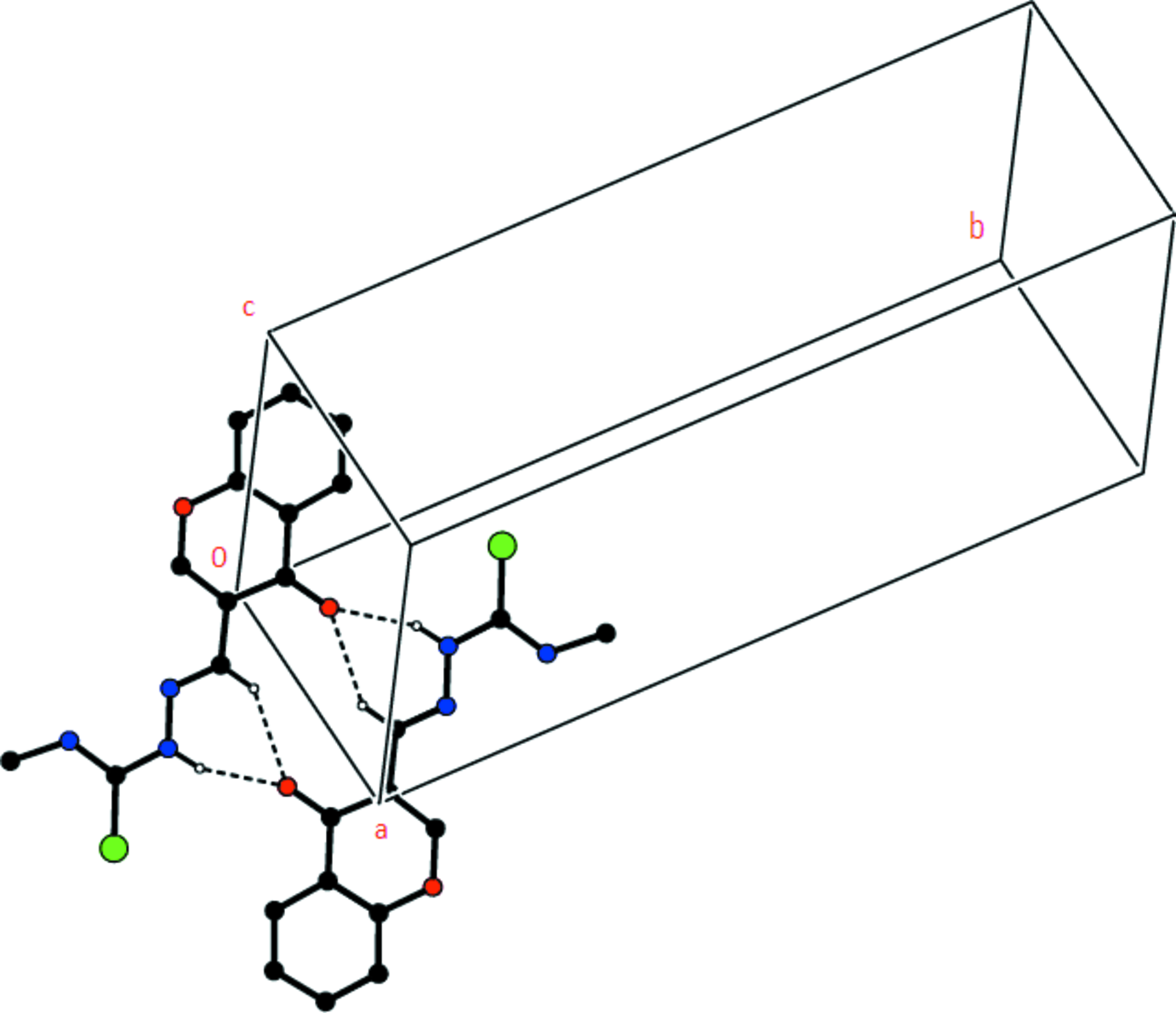

Supplement: Supplementary file 5 [file e-70-o1151-fig2.tif]
